# A Massive Green Tide in the Yellow Sea in 2021: Field Investigation and Analysis

**DOI:** 10.3390/ijerph191811753

**Published:** 2022-09-17

**Authors:** Minjie Song, Fanzhou Kong, Yifan Li, Jin Zhao, Rencheng Yu, Mingjiang Zhou, Peng Jiang, Tian Yan

**Affiliations:** 1 CAS Key Laboratory of Marine Ecology and Environmental Sciences, Institute of Oceanology, Chinese Academy of Sciences, Qingdao 266071, China; 2 University of Chinese Academy of Sciences, Beijing 100049, China; 3 Laboratory for Marine Ecology and Environmental Science, Pilot National Laboratory for Marine Science and Technology, Qingdao 266071, China; 4 Center for Ocean Mega-Science, Chinese Academy of Sciences, Qingdao 266071, China; 5 CAS Key Laboratory of Experimental Marine Biology, Institute of Oceanology, Chinese Academy of Sciences, Qingdao 266071, China

**Keywords:** green tide, Yellow Sea, development, causes

## Abstract

A massive green tide occurred in the Southern Yellow Sea (SYS) in 2021. As in previous years, its high biomass caused trouble to the coastal environment and landscape in 2021. Unusually, the 2021 green tide was unexpectedly massive. Thus, field surveys and remote sensing were conducted in the SYS from December 2020 to July 2021. Compared to pure satellite observations, it revealed the initial development of the green tide more clearly. Given the effects of temperature and nutrient changes on green tide biomass, we compared the environmental parameters in recent years and found no significant increase in nutrient concentrations or changes in temperature of the SYS in 2021. Relative to 2020, the 2021 green tide exhibited a wide distribution, high biomass, and prolonged duration. It was mainly affected by (1) reduced implementation of source-control measures and (2) limited *Sargassum* biomass, which reduced competition for *Ulva prolifera*. Strengthening the implementation of source control measures in Subei Shoal is recommended, which is currently the most effective way to control green tides.

## 1. Introduction

Large-scale green tides have recurred in the Southern Yellow Sea (SYS), China, for the past 15 years, since 2007, causing serious damage to tourism and aquaculture industries in coastal Shandong and Jiangsu Provinces and threatening ecosystem health in the SYS [1,2]. Although non-toxic in itself, green algae can produce hydrogen sulfide (H_2_S) and other toxic gases during bulk deposition and decay along the coast [3]. It is estimated that the total economic loss caused by green tides in 2008 was about RMB 1.3 billion [3].

During systematic research on the formation mechanism, key underlying processes, and prevention strategies of green tides in the SYS, multiple methods have been used, such as in situ investigation, satellite remote sensing, and molecular identification. The causative species of large-scale green tides in the SYS is the ‘floating’ ecotype of *Ulva prolifera*, mainly distributed in Subei Shoal [4,5,6] and later identified as *U. prolifera* subsp. qingdaoensis [7]. Cultivation of the red alga *Neopyropia* and eutrophication in Subei Shoal play important roles in the growth and transformation of *U. prolifera* from its attached to floating form [8,9]. Then, wind and tidal currents contribute to the northward drifting of floating green macroalgae from Subei Shoal [10,11].

Source-controlling measures and prevention strategies have been proposed based on the green tide formation mechanism and the alga’s key developmental processes [12]. Effective mitigation was implemented in spring 2018 by collecting algae at the source area, thus providing a suitable marine environment for the success of the Shanghai Cooperation Organization Summit in Qingdao [13]. In 2020, a large-scale field trial was conducted to eliminate attached green macroalgae from the entire *Neopyropia* culture region in Subei Shoal [14], resulting in the smallest green tide relative to that experienced in the previous ten years [15] and reflecting the effectiveness of source-controlling measures. 

However, a so-called ‘largest recorded’ green tide occurred again in the SYS in 2021. Its distribution area (61,898 km^2^ on June 21) is the largest recorded to date, placing tremendous demand for green tide control on coastal cities of the southern Shandong Peninsula [15]. Over 450,000 tons of algal biomass were collected, and ca. 12,686 fishing vessels were deployed from Qingdao until July 12 (data from China News). The causes and impacts of this large-scale green tide attracted a great deal of public interest and attention. Doubts were also raised about an adequate understanding of the formation mechanism of green tides and the usefulness of the source-controlling measures adopted in Subei Shoal during these blooms.

This study reports the results of field studies and satellite image analysis aimed at determining the species, biomass, and distribution of attached/detached green macroalgae and floating *Sargassum* in the SYS (including Subei Shoal) from December 2020 to July 2021. Comprehensive analyses of environmental conditions and the influence of mitigation actions to reduce green tide in the SYS in 2021 were conducted. Comparative data obtained over the years on the source, processes, nutrient concentrations, sea surface temperature (SST), and other factors contributing to green tides are also summarized and discussed, and additional green tide prevention and mitigation strategies in the SYS are proposed. 

## 2. Materials and Methods

This study combined field investigation and satellite remote sensing. Cruise studies of floating macroalgae and green algae attached to the *Neopyropia* rafts were carried out in Subei Shoal in December 2020 and February–March 2021. A comprehensive investigation of macroalgal species and their spatial distribution were carried out in Subei Shoal and the SYS in April and July 2021, respectively. Additionally, Sentinel-3 remote sensing images were used to track the green tide in the SYS from May to July 2021.

### 2.1. Cruise Data

#### 2.1.1. Visual Observations

During the cruises, the biomass of floating macroalgae was recorded every 30 min from the area of floating patches within sight. The classification criteria were as follows:

Level 0: no floating patches.
Level 1: patch area less than 5 m^2^.
Level 2: patch area between 5 and 250 m^2^.
Level 3: patch area between 250 and1000 m^2^.
Level 4: patch area between 1000 and 25,000 m^2^.
Level 5: patch area between 0.025 and 1 km^2^.
Level 6: patch area greater than 1 km^2^.

#### 2.1.2. Biomass Quantification

The floating algal biomass was assessed by trawling. Briefly, a plankton nylon net (1 cm mesh size and 1 m diameter) was towed horizontally to collect the floating biomass. The vessel moved at a low speed (2–3 knots) for 10–15 min to allow the net to tow through a given area of surface water. The average biomass (in tons km^−2^) at each station was then calculated as the total wet weight of the sampled macroalgae divided by the trawling area [16]. For the biomass containing a mixture of both algae (*Ulva* and *Sargassum*), these were separated and weighed individually. The total floating biomass was computed as the average biomass across all the surveyed stations multiplied by the total survey area. The samples were transported back to the laboratory at 4 °C for further analysis. 

#### 2.1.3. Species Identification

Genomic DNA was extracted from individual thallus using a modified CTAB method [17]. Then, all the specimens were identified by their ITS (internal transcribed spacer) sequences [18]. Morphological features of the samples were also analyzed to separate *Ulva linza* and *U. prolifrea* for the LPP (*U. linza-procera-prolifera*) clade [19], which was further confirmed by the 5S rDNA spacer sequences [20]. To elucidate the relationship among the algae samoles, 17 ITS sequences from Ulva and Blidingia were downloaded from GenBank as references. Phylogenetic trees were built using Maximum Likelihood (ML) methods with MEGA (10.0). Bootstrap value of 500 was taken in the tree construction.

#### 2.1.4. Nutrient Analysis

Water samples were collected during surveys of thirteen sample sites in Subei Shoal in April and about twenty sample sites in the SYS in July 2021, as well as in 2019 and 2020. The collected water was immediately filtered through a 25 mm GF/F glass filter membrane (pore size 0.68 µm) at a pressure below 50 kPa. Then, the concentrations of dissolved inorganic nitrogen (DIN) (nitrate, NO_3_^−^-N; nitrite, NO_2_^−^-N; and ammonium, NH_4_^+^-N), dissolved inorganic phosphorus (DIP) were determined from the filtered water using a SKALAR sa3000/5000 chemistry unit (SKALAR Company, Breda, The Netherlands); the details of the protocol were described by Wang et al. [21].

### 2.2. Remote Sensing Data

#### 2.2.1. Floating Algal Index (Sentinel-3)

The Ocean and Land Color Instrument (OLCI) aboard Sentinel-3 level-1 (since 2016, https://developers.google.com/earth-engine/datasets/catalog/COPERNICUS_S3_OLCI#description, accessed on 29 July 2021) provide observations in the visible–near-infrared domain from 0.4 to 1.0 µm with a pixel size of 300 m. In this study, the false color composite used band 17 (865 nm, near the infrared band), band 6 (560 nm, green), and band 3 (442.5 nm, blue), making floating algae obvious in shades of red, since vegetation reflects a large portion in near-infrared light, such that the brighter the red, the healthier the floating alga. The data were retrieved from the Google Earth Engine (GEE) platform. Images were generated by the code (https://code.earthengine.google.com/a3958318311b0ab66a66fec2dd5a883d, accessed on 29 July 2021).

#### 2.2.2. SST Data

Monthly SST data were derived from the global hybrid coordinate ocean model (HYCOM) [22]. To test whether there are differences in water temperature between 2021 and previous years, monthly averaged SSTs for Subei Shoal (32° N–34° N, 120° E–122° E) and the SYS (33° N–37° N, 119° E–123° E) from April to July 2016–2021 were calculated in ArcGIS 10.8 (https://www.arcgis.com/, accessed on 29 March 2022) with zonal statistics.

### 2.3. Statistical Analysis

Statistical analysis of these data was performed using IBM SPSS Statistics 26 (IBM, Armonk, NY, USA). The significance levels based on the two-tailed *p*-values were measured using a Student’s *t*-test.

## 3. Results

### 3.1. Floating Macroalgal Distribution in Subei Shoal from Winter 2020 to Early Spring 2021

**Figure 1:**
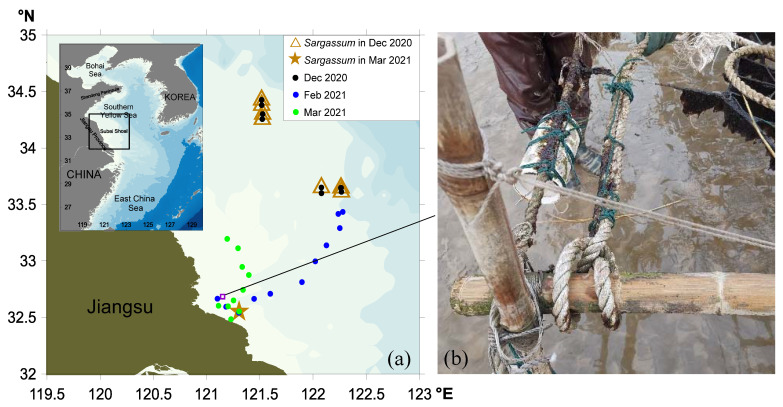
Cruise observation stations in Subei Shoal, Southern Yellow Sea, in December 2020 (indicated by black circles), February (blue circles), and March (green circles) in 2021. (**a**) The location of *Sargassum*, indicated by brown symbols, discovered in December 2020 (triangles) and March 2021 (star). The purple open square marks the location of the *Neopyropia* cultivation rafts (**b**).

**Figure 2:**
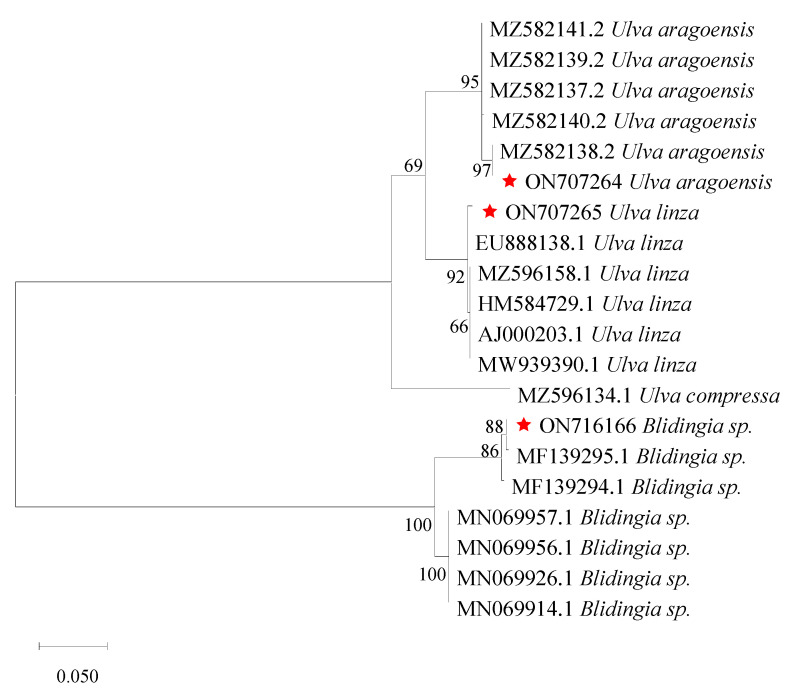
ML phylogenetic tree based on ITS sequences. The sequences with a red star were amplified from field samples.

The biomass of green macroalgae in Subei Shoal was relatively low from December 2020 to March 2021 (Figure 1). Field studies found that there was limited biomass of green algae on the cultivation ropes (Figure 1b). Based on morphological characteristics, the dominant species were identified as *Blidingia minima*, *Ulva aragoensis*, and *Ulva linza*, being consistent with sequence analysis results of the ribosomal ITS region and 5S region (Figure 2). The ITS and 5S sequences of the samples used in this study have been submitted to GenBank (https://www.ncbi.nlm.nih.gov/genbank/, accessed on 11 June 2022), and their accession numbers were ON707264, ON707265, ON716166, and ON734047. Meanwhile, no floating green macroalgae were observed in Subei Shoal, while floating *Sargassum* was found in offshore waters in December 2020 and in coastal waters in March 2021 (Figure 1a).

### 3.2. Floating Macroalgal Biomass in Subei Shoal in Spring 2021

**Figure 3:**
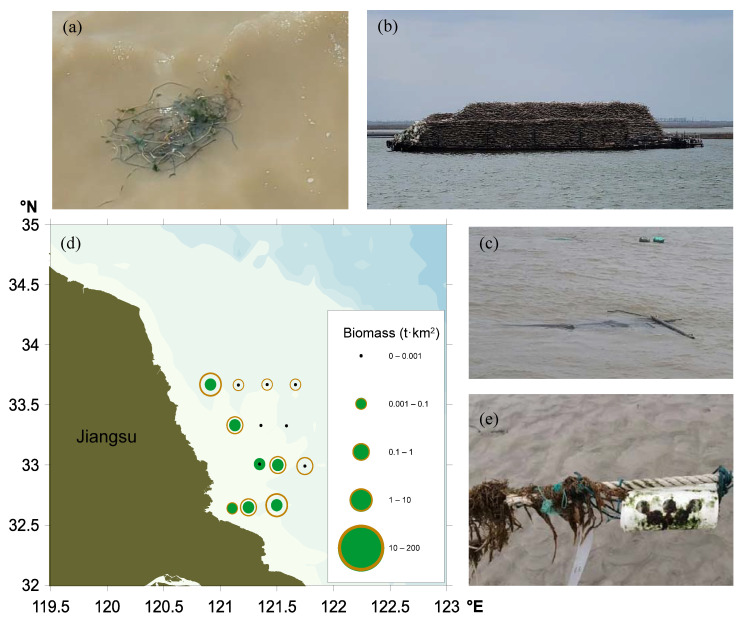
Biomass of floating *Ulva* (green filled circles) and *Sargassum* (brown open circles) in Subei Shoal in April 2021. (**a**) Floating green macroalgae attached to floating pieces of nursery net; (**b**) rafts collected on a boat; (**c**) scattered remnants of rafts floating in the sea; (**d**) *Sargassum* accumulated in *Neopyropia* mariculture rafts; (**e**) floating biomass of *Ulva* (green circles) and *Sargassum* (brown circles) assessed by a trawling survey.

The biomass of green macroalgae in Subei Shoal began to increase in April 2021 (shown below). A trawling survey indicated that the floating biomass of green macroalgae averaged 0.01 t km^−2^ in this area and was mostly attached to floating pieces of nursery net (Figure 3a). The ‘floating’ ecotype of *Ulva prolifera* was detected within these green macroalgae, as well as *U. flexuosa*, *U. linza,* and *Ulva compressa* (unpublished data). At the time, the mariculture rafts were being collected (Figure 3b), with some of these rafts left in the sea (Figure 3c). These results indicate that green macroalgae attached to the *Neopyropia* rafts began to enter the sea in April 2021 during the early floating stage. 

The floating biomass of *Sargassum* was relatively high (0.58 t km^−2^ on average) compared to that of green macroalgae (Figure 3d). These scattered patches (<1 m^2^) of *Sargassum horneri* could occasionally form strips at the surface, and they could be observed attached to the *Neopyropia* rafts (Figure 3e).

### 3.3. Green Tide in the SYS in Late Spring and Summer 2021

**Figure 4:**
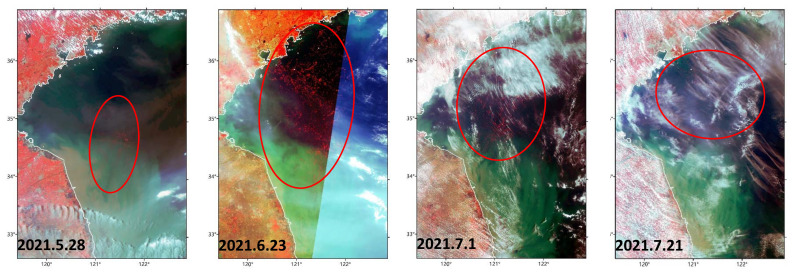
Satellite images of floating green macroalgae in the Southern Yellow Sea from 28 May to 21 July 2021 (false-red-green-blue (RGB) composite of Ocean and Land Color Instrument (OLCI) images, R: band 17, G: band 6, B: band 3) (red circles indicate the main distribution area of floating biomass).

Floating *Ulva* drifted northward from Subei Shoal to nearshore waters of the Shandong Peninsula during May–July 2021 (Figure 4). Massive floating biomass was observed drifting out of Subei Shoal by satellite in late May (https://ovl.oceandatalab.com/?date=1621857600000&timespan=1d&zoom=9&extent=13303597.15233_3931589.12868_13709324.898412_4139497.8455868&products=3857_Sentinel-3_OLCI_true_RGB%213857_Sentinel-3_OLCI_NIR_BC&opacity=100_100&stackLevel=85_86.01&selection=11&center=13506461.025371_4035543.4871334, accessed on 18 August 2022) and then observed to spread throughout open SYS waters on June 23. This floating biomass continued to drift northward and expand until it landed on the south coast of the Shandong Peninsula in June and was still massive in scale on July 21 or even later (shown in the platform above).

### 3.4. Floating Macroalgae in the SYS in Summer 2021

**Figure 5:**
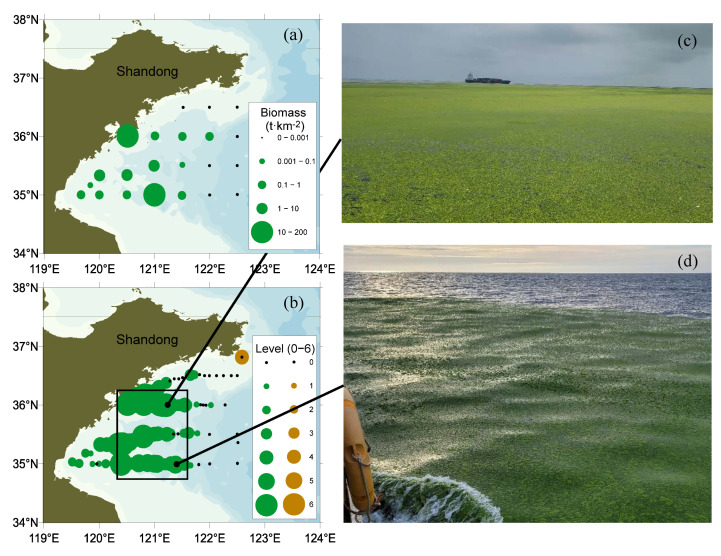
Distribution and biomass of floating *Ulva* (green circles) and *Sargassum* (brown circles) in the Southern Yellow Sea (SYS) in July 2021. (**a**) Floating biomass and distribution assessed by trawling; (**b**) biomass levels and distribution assessed by visual observation; (**c**,**d**) field images in the survey region.

The SYS green tide in July 2021 was characterized by a large distribution area and high biomass. It was further investigated during 4–9 July 2021, using a combination of trawling and continuous visual observation. The distribution of biomass density at each station, which peaked at a maximum of 170 t wet weight km^−2^, is shown in Figure 5a. When calculated over the 40,000 km^2^ sea area surveyed, the total biomass of floating green macroalgae was ca. 389,000 tons (swept area method [16]).

This massive algal biomass aggregated in the sea area 35° N–36° N, 120°15′ E–121°30′ E (Figure 5b), and several huge patches (>1 km^2^) were found in the sea area near Qingdao. The floating *Ulva* along the above sector showed an overall yellow-green color (Figure 5c) when some of the green algae began to decompose. High biomass was also observed along the 35° N sector distributed in strips and with a patchy distribution, characterized by an emerald green color (Figure 5d).

The floating biomass consisted mainly of green macroalgae. The floating green macroalgae were identified as the ‘floating’ ecotype of *Ulva prolifera* based on morphological features, which was further proved by molecular identification (unpublished data). Almost no *Sargassum* was observed in the SYS, and this was only detected in waters adjacent to the southeastern Shandong Peninsula (Figure 5b). 

### 3.5. Characteristics of Ulva Prolifera Green Tide in the SYS in 2021

Compared with the green tides occurring in the SYS in previous years, the formation and development of the 2021 green tide showed common characteristics as follows: 1. the same original source area; satellite images, cruise observations, and molecular identification all indicated that floating patches of *U. prolifera* in the SYS in 2021 originated from Subei Shoal; 2. green algae experienced a common fate, i.e., SYS green tides in 2021 and previous years first appeared in Subei Shoal and subsequently drifted northward from the source area to form massive green tides in the Southern Yellow Sea; 3. they had the same causative species, namely the ‘floating’ ecotype of *U. prolifera*. 

**Figure 6:**
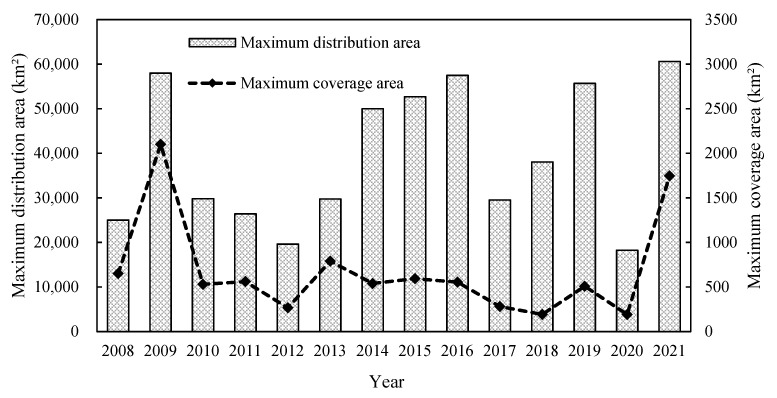
Maximum daily distribution area (bar graph) and maximum coverage area (dashed line) of *Ulva prolifera* green tides between 2008 and 2021 (data from The Bulletin of China Marine Disaster: 2009–2021 (MNR, 2009–2021)). Notes: Data based on satellite remote sensing (250 m and 50 m resolution). The distribution area is equal to the sum of the coverage area and the gap between the floating patches.

The main difference between the SYS green tide in 2021 and in previous years was that in 2021, it was characterized by a relatively wider distribution area, high biomass, and prolonged duration (Figure 6). Additionally, little *Sargassum* was observed in the SYS in April and July 2021.

### 3.6. Factors Influencing the Scale of the 2021 Green Tide in the SYS

**Figure 7:**
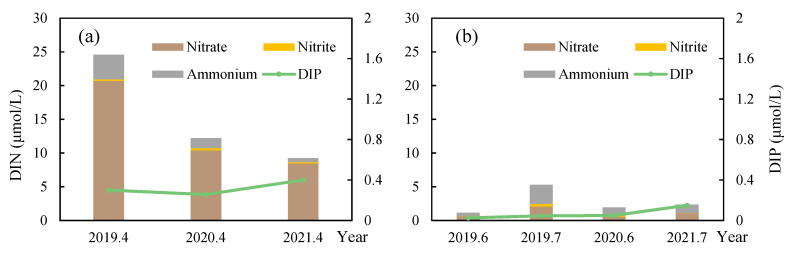
Average nutrient concentrations of Subei Shoal in April (**a**) and the Southern Yellow Sea in June and July (**b**) in 2019–2021. DIN: dissolved inorganic nitrogen (including nitrate, NO_3_^−^–N; nitrite, NO_2_^−^–N; and ammonium, NH_4_^+^–N); DIP: dissolved inorganic phosphorus.

**Figure 8:**
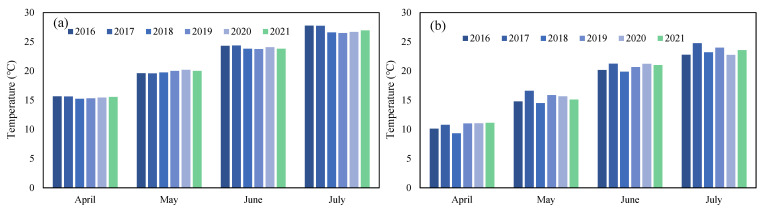
Monthly averaged sea surface temperature of Subei Shoal (**a**) and the Southern Yellow Sea (**b**) from April to July 2016–2021.

The scale of the 2021 green tide was affected by many factors, which may include nutrient concentrations, temperature, and *Sargassum* biomass in the SYS. Nevertheless, nutrient concentrations in 2021 were not the highest in recent years (Figure 7). Moreover, there is a decreasing trend of nutrient concentrations in Subei Shoal in April 2019–2021 (Figure 7). In terms of temperature, whether it is Subei Shoal or the SYS, the monthly average sea surface temperature in 2021 was not significantly different from the previous years (Figure 8), but the *Sargassum* biomass in 2021 was the lowest in the recent three years, which may be a possible reason of the massive green tide in 2021.

## 4. Discussion

### 4.1. Analysis of the Causes of SYS Green Tide in 2021

There were various assumptions about the causes of the massive green tide in 2021. Maybe the 2021 green tide has a different source. However, no floating green algae were discovered during the cruise investigations in the SYS in April 2021, except for Subei Shoal. Algal blooms are always related to nutrient enrichment, and Wang and Wu [23] thought a large number of nutrient inputs were the reason for the massive green tide in 2021, but there is no increasing trend of nutrient concentrations in Subei Shoal in April 2019–2021 (Figure 7). The nutrient concentrations in Subei Shoal, even in 2021, were nearly ten times higher than that of the L1 medium, which is commonly used in algae culture in laboratory. It means that it is always rich enough for algae to grow. Similarly, *U. prolifera* can reach a high growth rate (16.7% d^–1^) under suitable temperature [24], which Zheng et al. [25] thought could be an important impact factor. Taking this into account, we analyzed the SST in the SYS from April to July in the recent six years and found no significant differences (*p* > 0.05) (Figure 8). The causes of SYS green tide in 2021 may be as follows:

Firstly, the massive green tide in the SYS in 2021 may be attributable to the inadequate removal of *Neopyropia* mariculture rafts caused by extreme weather conditions. Many extreme weather events occurred in the region in spring 2021, such as the severe convective weather that affected Jiangsu Province on April 30 [15,26]. As reported by Qingdao Daily on July 30, extreme weather events involving gales and rainstorms during May and June 2021 may result in the re-entry at sea of culture rafts collected on the shore, after which about 130,000 bamboo poles were salvaged from the SYS (unpublished data). *Ulva prolifera* attached to these floating rafts became the primary source of the floating green tide, which could proliferate rapidly in Subei Shoal. 

Secondly, the green tide may have developed due to reduced algal-removal action and supervision in Subei Shoal. Following the practice implemented in 2018, ca. 3000 tons of the initial floating algal biomass were collected from Subei Shoal and adjacent coastal waters [14]. As a result, the maximum coverage area in 2018 was the lowest recorded since 2013 (Figure 6), while the initial floating biomass was much greater than that in previous years [14,27]. Combined with acid treatment, *Neopyropia* cultivation in Subei Shoal in 2020 ended one month earlier than during regular operations, and all the rafts were removed from the shoal (without any macroalgae being released) by May 8 [13]. As a result of all the source-controlling measures, the size of the green tide in 2020 was the smallest, occurring over the previous ten years (Figure 6). Nevertheless, despite the algal-removal measures such as acid treatment and other early removal measures applied in Subei Shoal, raft collecting and green algae salvaging may have been constrained in May 2021 due to the extreme weather or/and other factors.

**Figure 9:**
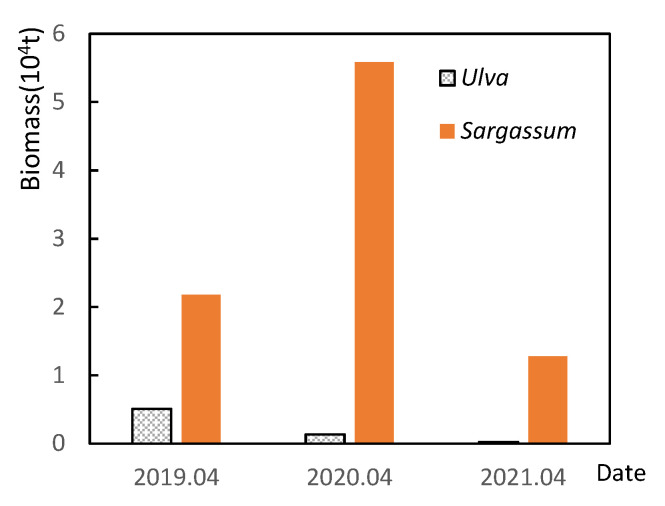
Biomass of floating *Ulva* and *Sargassum* in Subei Shoal in April 2019–2021.

Finally, limited *Sargassum* was available in 2021 to compete for space and nutrients, which is consistent with the view of Li et al. [28]. Laboratory studies have found that *S. horneri* decomposition and the culture medium can inhibit the attachment and germination of microscopic propagules of *U. prolifera* at high concentrations but can promote them at low concentrations [29]. Field observation and remote sensing data showed that a large-scale *S. horneri* golden tide (maximum distribution of 160,000 km^2^) entered Subei Shoal in 2017 [30], a year when the size of the green tide was smaller than in the previous three years (Figure 6), suggesting some interactions between the two macroalgal species [2,31]. Almost no *Sargassum* was observed in the SYS in July 2021 (Figure 5b), and its biomass was also low in Subei Shoal in spring 2021 compared to that in previous years (Figure 9). This may have promoted the outbreak of the so-called “greatest recorded green tide” in the SYS.

### 4.2. Prevention Strategies and Recommendations to Control Green Tides in the SYS

Continued strengthening of prevention measures for green algae in the source region is necessary. *Ulva prolifera* grows and spreads rapidly once it drifts away from Subei Shoal [14]. Control measures in open waters are costly and ineffective. Strategies to control the green algal source nearshore are believed to be more efficient and cost-effective than those feasible offshore. These measures were inefficiently implemented in 2021 compared to 2020, thus presumably resulting in the massive green tide experienced in the SYS in 2021.

The critical period during the early development of green tides, from late April to May, should be focused on for algal removal. Once missed, a few tons of initial floating biomass can proliferate into thousands of tons of macroalgal mats within one or two months. It is thus critical for effective source control to reduce the biomass of green macroalgae attached to the *Neopyropia* rafts during this key period. Control measures applied during the early development of green tides, such as acid treatment and algal collection, worked well in Subei Shoal in 2020 [13]. In contrast, source-control measures in Subei Shoal may have proved inadequate when disrupted by extreme weather conditions in spring 2021. 

The removal of the *Neopyropia* rafts from Subei Shoal before late April should be encouraged. The rafts, including the nursery nets, bamboos, and connecting ropes, can be removed from the shoal without any major cleaning effort, rather than abandoning them haphazardly onshore to prevent their accidental re-entry at sea during extreme weather.

## 5. Conclusions

In conclusion, this study demonstrated the complete development process of the 2021 massive green tide mainly based on field investigations. By comparing the survey data in previous years, we found that the massive green tide was mainly affected by (1) reduced implementation of source-control measures and (2) limited *Sargassum* biomass, which reduced competition for *Ulva prolifera*. Strengthening the implementation of source control measures in Subei Shoal is recommended, which is currently the most effective way to control green tides.

## Data Availability

The data can be obtained from the corresponding authors upon reasonable request.
